# Effect of surface vacancies on the adsorption of Pd and Pb on MgO(100)

**DOI:** 10.1007/s00706-018-2159-1

**Published:** 2018-02-13

**Authors:** Piotr Matczak

**Affiliations:** Department of Physical Chemistry, Faculty of Chemistry, University of Łódź, Pomorska 163/165, 90-236 Lodz, Poland

**Keywords:** Quantum chemical calculations, Metals, Surface, Heterogeneous catalysis, Defects

## Abstract

**Abstract:**

Theoretical quantum mechanical calculations have been carried out to establish the effect of surface vacancies on the adsorption of Pd and Pb atoms on the defective MgO(100) surface. The investigated defects included neutral, singly and doubly charged O and Mg vacancies on the (100) surface of MgO. These vacancies played the role of F_s_^*n*+^ and V_s_^*n*−^ (*n* = 0, 1, 2) adsorption centers for a single Pd or Pb atom. From the results of calculations, it is clear that the Pd- and Pb-atom adsorption at the F_s_^*n*+^ and V_s_^*n*−^ centers shows different characteristics than at the regular O^2−^ and Mg^2+^ centers. Drastic changes in geometric, energetic, and electronic parameters are evident in Pd/V_s_^*n*−^ and Pb/V_s_^*n*−^. The effect of F_s_^0^ and F_s_^+^, which in practice are the most important vacancies, is smaller, yet the adsorption of Pd and Pb at these centers is more energetically favorable than at the regular O^2−^ center. Of the two metals studied, the atom of Pd is bound by the F_s_^0^ and F_s_^+^ centers with higher adsorption energies.

**Graphical abstract:**

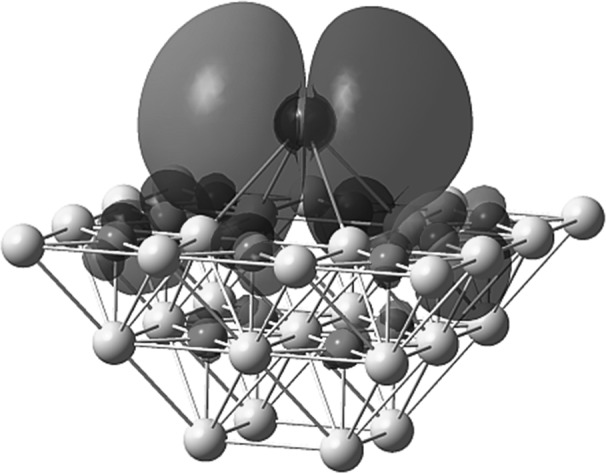

**Electronic supplementary material:**

The online version of this article (10.1007/s00706-018-2159-1) contains supplementary material, which is available to authorized users.

## Introduction

Palladium supported on oxides has found numerous applications in heterogeneous catalysis [1–3]. The catalytic performance of Pd/oxide systems can be improved by coupling their Pd part with another metallic element [4, 5]. In the resulting bimetallic Pd-M/oxide catalysts, Pd is usually combined with a typical metal or a half metal (M=Al, Si, Zn, Ga, Ge, In, Sn, Sb, Te, Tl, Pb, or Bi) [5]. Recently, it has been reported that bimetallic Pd–Pb/MgO catalysts are more effective than monometallic Pd/MgO catalysts in performing aerobic oxidations of amines [6] and oxidative esterification of methacrolein with methanol [7, 8]. Understanding the enhanced catalytic performance of these bimetallic catalysts requires a detailed knowledge of several fundamental aspects of their metal-oxide interfaces. These aspects include, in particular, geometric and electronic features of interfaces and the strength of metal-oxide interaction. Ideally, the first step of such a characterization should concern small clusters of Pd and Pb, or even better single Pd and Pb atoms, at individual adsorption sites on a well-defined single-crystal MgO surface, such as the MgO(100) one. This surface is often regarded as a prototypical oxide surface in studies of metal adsorption, because it has a simple structure and well-defined stoichiometry [9]. Additionally, it is relatively easy to form defects on this surface [10].

Various experimental techniques can yield information on the structure and energetics of metal nanoparticles and films deposited on oxides [11] and many experimental efforts have indeed been undertaken to characterize both Pd/MgO(100) [12–17] and Pb/MgO(100) [18–22]. On the other hand, properties of single atoms adsorbed on oxide surfaces are available mostly from theoretical investigations based on computational quantum mechanical approaches [23]. Of Pd and Pb on MgO(100), only the former has become a subject for a large number of theoretical studies of single metal atom adsorption so far [15, 24–37]. To the best of our knowledge, no theoretical quantum mechanical investigations of Pb/MgO(100) have been reported until now.

This work is aimed at providing a theoretical quantum mechanical description for the adsorption of Pd and Pb on the MgO(100) surface with various point defects. Both oxygen (F_s_^*n*+^) and magnesium (V_s_^*n*−^) vacancies in three charge states (*n* = 0, 1, 2) have been taken into account. From experimental studies [38, 39], it is known that such defects may be formed on MgO(100), but with a significant differentiation in their concentrations. What is particularly important is that various defects occurring on the MgO(100) surface can act as anchoring sites for metal nanoparticles [38, 40], and additionally, they can modify the properties of deposited metal nanoparticles [38, 41]. Here, a set of essential geometric, energetic and electronic parameters for a single Pd or Pb atom adsorbed at the F_s_^*n*+^ and V_s_^*n*−^ centers has been calculated to characterize the fundamental aspects of Pd- and Pb-atom adsorption on defective MgO(100). Due to the lack of any previous theoretical studies for Pb/MgO(100), it is vitally important to provide an insight into the effect of surface vacancies on Pb-atom adsorption at atomic level.

## Results and discussion

The calculated values of three essential parameters (height from the surface *h*, adsorption energy *E*_ads_ and electron charge *q*) characterizing a single Pd atom adsorbed at the F_s_^*n*+^ and V_s_^*n*−^ centers on the defective MgO(100) surface are listed in Table 1. It is evident that the Pd-atom adsorption at the F_s_^*n*+^ centers is far different from that at the V_s_^*n*−^ centers. The heights of the adsorbed Pd atom from the F_s_^*n*+^ centers are much greater than the *h* values of Pd/V_s_^*n*−^ structures. The Pd atom is almost inserted into the vacancy cavity for Pd/V_s_^0^ and Pd/V_s_^−^ in their high-spin (HS) states. The smaller *h* values of Pd/V_s_^*n*−^ are accompanied by the highly exothermic *E*_ads_ energies. The comparison of *E*_ads_ for the low-spin (LS) and HS states reveals that, with the possible exception of Pd/V_s_^2−^, all remaining Pd/F_s_^*n*+^ and Pd/V_s_^*n*−^ structures prefer their LS state. In other words, the adsorbed Pd atom tends to conserve its spin state (the ground state of free Pd atom exhibits a singlet multiplicity). The preference of LS state is particularly noticeable for Pd/F_s_^+^ because its HS state does not lead to any energetic stabilization. In the case of Pd/V_s_^2−^, its LH and HS states lie very close to each other and it is difficult to appoint the ground state with absolute certainty. The *E*_ads_ energy shows a clear dependence on the formal charge (*n*) of vacancies. The Pd-atom adsorption at F_s_^*n*+^ becomes less and less exothermic in the order F_s_^0^ > F_s_^+^ > F_s_^2+^. The same sequence can be observed for Pd/V_s_^*n*−^. The values of *q* acquired by the adsorbed Pd atom indicate that it behaves as an electron acceptor when it sits at the F_s_^*n*+^ centers. The Pd/F_s_^0^ structure demonstrates the greatest charge transfer to the metal atom. This is because the isolated F_s_^0^ center possesses two extra electrons that are largely localized in its cavity [42] and a significant amount of this electron charge can be easily transferred to an adsorbed atom [34]. Unlike Pd/F_s_^*n*+^, the Pd/V_s_^*n*−^ structures show the opposite direction of charge transfer. Our calculations predict that the charge transfer from the Pd atom to the V_s_^*n*−^ centers never exceeds 0.9 e even if the Pd atom is almost inserted into the V_s_^*n*−^ cavity. This charge transfer and the small *h* heights lead to a significant electrostatic stabilization between the ionized Pd atom and the V_s_^*n*−^ centers.

**Table 1:** Essential parameters characterizing the adsorption of a single Pd atom at various centers on the defective MgO(100) surface

Center	*h*^LS^/Å	*h*^HS^/Å	*E*_ads_^LS^/eV	*E*_ads_^HS^/eV	*q*^LS^/e	*q*^HS^/e
F_s_^0^	1.540 (1.539)	1.708 (1.726)	3.85 (3.78)	1.65 (1.57)	− 1.521 (− 1.525)	− 1.536 (− 1.522)
F_s_^+^	1.504 (1.503)		2.53 (2.45)		− 0.871 (− 0.873)	
F_s_^2+^	1.509 (1.505)	1.706 (1.713)	1.37 (1.27)	0.29 (0.26)	− 0.261 (− 0.264)	− 0.239 (− 0.234)
V_s_^0^	0.383 (0.382)	0.160 (0.160)	7.58 (7.59)	7.29 (7.31)	0.795 (0.810)	0.888 (0.894)
V_s_^−^	0.601 (0.589)	0.157 (0.133)	5.42 (5.40)	4.63 (4.98)	0.437 (0.448)	0.880 (0.893)
V_s_^2−^	0.366 (0.331)	0.584 (0.538)	4.39 (4.70)	4.52 (4.66)	0.799 (0.782)	0.427 (0.433)
O^2−^	2.165 (2.148)	2.351	1.34 (1.30)	0.28	− 0.231 (− 0.233)	− 0.193
Mg^2+^	2.633 (2.636)		0.47 (0.39)		− 0.075 (− 0.082)	

Results obtained from calculations in which the Pd atom was described by the LANL08(f) basis set are listed without parentheses, whereas the results from calculations utilizing the def2-TZVP basis set for Pd are in parentheses

Results for centers with the unbound Pd atom (*E*_ads_ < 0) are not presented

It is instructive to compare the Pd-atom adsorption at the vacancies with that occurring at non-defective sites. Results describing the adsorption of a single Pd atom at the regular anionic O^2−^ and cationic Mg^2+^ centers of defect-free MgO(100) surface are appended to Table 1. As evidenced by the *E*_ads_ values of Pd/O^2−^ and Pd/Mg^2+^, the Pd atom binds preferentially to the O^2−^ center in the LS state. A small charge transfer to the metal atom appears for Pd/O^2−^, while the Pd atom remains essentially neutral at the Mg^2+^ center. The Pd-atom adsorption at O^2−^ is less energetically favorable than at F_s_^0^ and F_s_^+^. The Pd/O^2−^ structure also demonstrates a larger *h* value compared to those of Pd/F_s_^0^ and Pd/F_s_^+^. On the other hand, the Pd/O^2−^ and Pd/F_s_^2+^ structures are formed with very similar *E*_ads_ energies, although the former exhibits a much larger *h* value.

An inspection of the results in Table 1 also reveals that the kind of the basis set assigned to the Pd atom most often has a rather minor effect on the calculated values of *h*, *E*_ads_ and *q*. A discrepancy in the interpretation of the results obtained from LANL08(f) and def2-TZVP appears for Pd/V_s_^2−^ and Pd/O^2−^. The calculations employing the two basis sets designate different spin states as the energetically preferred state of Pd/V_s_^2−^. In the case of Pd/O^2−^ in the HS state, the calculations involving the LANL08(f) basis set predict an exothermic adsorption, in contrast to those carried out with def2-TZVP. However, the *E*_ads_^HS^ value obtained from LANL08(f) is actually quite close to zero, and therefore, the significance of this discrepancy should not be overemphasized.

Our findings made for the Pd-atom adsorption are essentially in good agreement with conclusions reported in previous experimental [14–16] and theoretical [15, 24, 26–28, 33, 35, 36] studies of Pd/MgO(100). It is well-known that the defect-free MgO(100) surface is generally rather unreactive toward the adsorption of metal atoms [43]. The Mg^2+^ centers exhibit particularly low reactivity toward metal atoms [25]. In consequence, Pd atoms preferably occupy the O^2−^ centers [28], with no significant charge transfer from or to the surface [27]. An experimental estimation of adsorption energy for Pd on MgO(100) is ca. 1.2 eV [14]. From an experimental measurement, a value of 2.22 Å was also deduced to be the height of an adsorbed Pd atom from the O^2−^ center [26]. Our *E*_ads_^LS^ and *h*^LS^ values for Pd/O^2−^ are very close to these experimental estimations. Similarly to metal adsorption on the defect-free MgO(100) surface, metal atoms on MgO(100) with defects also adsorb preferentially at centers where negative charge accumulates [33, 44]. More specifically, the F_s_^0^ centers play the key role in the adsorption of Pd atoms [15, 16]. This is because these centers are the main part of vacancies formed on MgO(100), which was confirmed both experimentally [10] and theoretically [45, 46]. Besides the F_s_^0^ centers, the F_s_^+^ centers can also occur, but they are less likely due to their large formation energy [42]. Even larger formation energy was determined for the F_s_^2+^ center [42]. Previous computational studies have shown that the Pd/F_s_^+^ interaction is weaker than the Pd/F_s_^0^ interaction but stronger than that of Pd/O^2−^ [15, 33, 36]. Apart from rendering this trend correctly, our *h* and *E*_ads_ values also reproduce quantitatively other theoretical results [15, 35, 36]. It has also been reported that the interaction between Pd and V_s_^*n*−^ centers is extremely strong [24]. According to an experimental study [10], the concentration of surface Mg vacancies seems, however, to be much lower than that of F_s_^0^ and F_s_^+^. Again, this is in line with large formation energies of V_s_^*n*−^ vacancies [42, 47].

This review of existing results for Pd/MgO clearly indicates that the computational methodology applied in this work leads to the correct description of Pd-atom adsorption on MgO(100) with surface vacancies. Thus, one can expect that the parameters characterizing the adsorption of Pb atom at the F_s_^*n*+^ and V_s_^*n*−^ centers should also be predicted reliably.

Essential parameters for the Pb atom adsorbed at the F_s_^*n*+^ and V_s_^*n*−^ centers are collected in Table 2. A careful inspection of these results reveals that there are several similarities between the Pd-atom adsorption and its Pb counterpart. The formation of Pb/V_s_^*n*−^ structures is associated with extremely exothermic *E*_ads_ values, many times greater than those calculated for the Pb/F_s_^*n*+^ structures. For Pb/F_s_^*n*+^, their *E*_ads_ energies decrease regularly with the growing formal charge of F_s_^*n*+^ center. The adsorption of Pb at V_s_^*n*−^ leads to a significant charge transfer from Pb to the V_s_^*n*−^ centers, while the reverse direction of charge transfer is observed for Pb/F_s_^0^ and Pb/F_s_^+^. A strong correlation between *E*_ads_ and the magnitude of charge transfer can be found for both the Pd/F_s_^*n*+^ and Pb/F_s_^*n*+^ structures. On the other hand, the Pb-atom adsorption turns out to be different in certain aspects from the Pd-atom adsorption. First, the large atomic radius of Pb causes this atom not to replace the missing Mg atom at the V_s_^*n*−^ cavity. The *h* values of Pb/V_s_^*n*−^ clearly indicate that the Pb atom sits higher above the V_s_^*n*−^ centers than it has been detected for Pd/V_s_^*n*−^. Second, the Pb atom easily becomes ionized, if adsorbed at the V_s_^*n*−^ centers, and the resulting charge transfer from Pb to these centers far exceeds one electron. The ionization potential of Pb is lower than that of Pd (7.42 eV [48] versus 8.34 eV [49]), thus the enhanced tendency of the former to donate electron charge to the V_s_^*n*−^ centers. The same direction of charge transfer yet much smaller in magnitude occurs for Pb/F_s_^2+^, whereas a negatively charged metal atom was found for Pd/F_s_^2+^. Third, the HS state is preferred for the Pb/F_s_^*n*+^ structures, which is a consequence of the triplet multiplicity of free Pb atom in its ground state. However, the extremely high *E*_ads_ values of Pb/V_s_^0^ and Pb/V_s_^−^ are sufficient for spin paring, and therefore, these structures favor the LS state. In the case of Pb/V_s_^2−^, the difference between its *E*_ads_^LS^ and *E*_ads_^HS^ energies is too small for spin quenching.

**Table 2:** Essential parameters characterizing the adsorption of a single Pb atom at various centers on the defective MgO(100) surface

Center	*h*^LS^/Å	*h*^HS^/Å	*E*_ads_^LS^/eV	*E*_ads_^HS^/eV	*q*^LS^/e	*q*^HS^/e
F_s_^0^	2.395 (2.331)	2.368 (2.340)	1.45 (1.66)	2.16 (2.33)	− 1.361 (− 1.363)	− 1.365 (− 1.376)
F_s_^+^	2.464 (2.422)	2.300 (2.282)	1.19 (1.32)	1.24 (1.37)	− 0.610 (− 0.630)	− 0.640 (− 0.660)
F_s_^2+^	2.671 (2.612)	2.797 (2.737)	0.65 (0.73)	0.81 (0.86)	0.172 (0.134)	0.162 (0.129)
V_s_^0^	1.028 (1.105)	0.606 (0.728)	9.55 (9.24)	5.85 (5.38)	1.226 (1.211)	1.215 (1.429)
V_s_^−^	1.005 (1.056)	0.571 (0.675)	7.04 (6.82)	4.01 (3.53)	1.215 (1.185)	1.544 (1.429)
V_s_^2−^	0.986 (1.026)	0.980 (1.009)	6.46 (6.22)	6.75 (6.58)	1.192 (1.156)	1.188 (1.158)
O^2−^	2.520 (2.562)	2.547 (2.576)	0.29 (0.33)	1.07 (1.11)	− 0.117 (− 0.140)	− 0.122 (− 0.133)
Mg^2+^		3.570 (3.464)		0.07 (0.09)		− 0.028 (0.003)

Results obtained from calculations in which the Pb atom was described by the LANL08d basis set are listed without parentheses, whereas the results from calculations utilizing the def2-TZVP basis set for Pb are in parentheses

Results for centers with the unbound Pb atom (*E*_ads_ < 0) are not presented

The kind of basis set assigned to metal atom affects the parameters of Pb-atom adsorption to a greater extent than the results for the Pd-atom adsorption. The greater discrepancies in the parameters obtained using LANL08d and def2-TZVP result from an inherent difference in the treatment of Pb atom with the two basis sets. These basis sets differ not only in the number of basis functions in their valence parts, but also in the size of their core parts treated with pseudopotentials. LANL08d is expected to yield less accurate results because (1) its quality is formally inferior to that of def2-TZVP and (2) a previous benchmark study confirmed its poorer performance [50]. Notwithstanding this difference, the application of either basis sets provides a qualitatively consistent picture of Pb-atom adsorption at the F_s_^*n*+^ and V_s_^*n*−^ centers.

To establish the effect of surface vacancies on the Pb-atom adsorption, Table 2 also shows the *h*, *E*_ads_, and *q* parameters calculated for Pb/O^2−^ and Pb/Mg^2+^. It is clear that the Pb-atom adsorption is possible only at the O^2−^ center on the defect-free MgO(100) surface. Similarly to Pb/F_s_^*n*+^, the Pb/O^2−^ structure tends to conserve the triplet multiplicity of Pb and its *E*_ads_^HS^ energy becomes more exothermic than *E*_ads_^LS^. On the other hand, the *E*_ads_^HS^ value for Pb/O^2−^ is smaller than those of Pb/F_s_^0^ and Pb/F_s_^+^. It proves that Pb atoms adsorb preferentially at the F_s_^0^ centers on the defective MgO(100) surface. It worth reminding here that, of the considered F_s_^*n*+^ and V_s_^*n*−^ vacancies, the F_s_^0^ centers are most abundant on the defective MgO(100) surface.

For the Pb/F_s_^*n*+^ structures, their propensity to change the spin state from HS to LS can be evaluated by calculating the difference between their *E*_ads_^HS^ and *E*_ads_^LS^ energies. The resulting HS → LS transition energies adopt smaller values than the excitation energy of a free Pb atom from its ground state to the lowest singlet state (0.95 and 0.89 eV at the B3LYP/LANL08d and B3LYP/def2-TZVP levels, respectively). Moreover, these transition energies are smaller than the HS → LS transition energy of Pb/O^2−^. It implies that the F_s_^*n*+^ centers facilitate the HS → LS transition in the adsorbed Pb atom.

An experimental study concerning the growth of Pb film on well-defined oxide surfaces [18] reported a calorimetrically measured initial heat of adsorption of 1.07 eV for Pb/MgO(100) at 300 K. This value was an average of the bonding of Pb atoms to MgO(100) and Pb–Pb bonding within small Pb nanoparticles formed on MgO(100). For such nanoparticles, their Pb–MgO(100) bond strength was roughly estimated to be either 0.33 or 0.16 eV, depending on the kind of Pb nanoparticles adsorbed (whether two- or three-dimensional Pb nanoparticles). In a more recent study based on atomic beam/surface scattering measurements [22], a range from 0.72 to 0.81 eV was proposed to be the heat of Pb adsorption at terrace sites on MgO(100). Our *E*_ads_^HS^ energy of Pb/O^2−^ exceeds by ca. 0.3 eV the upper limit of this range.

**Fig. 1:**
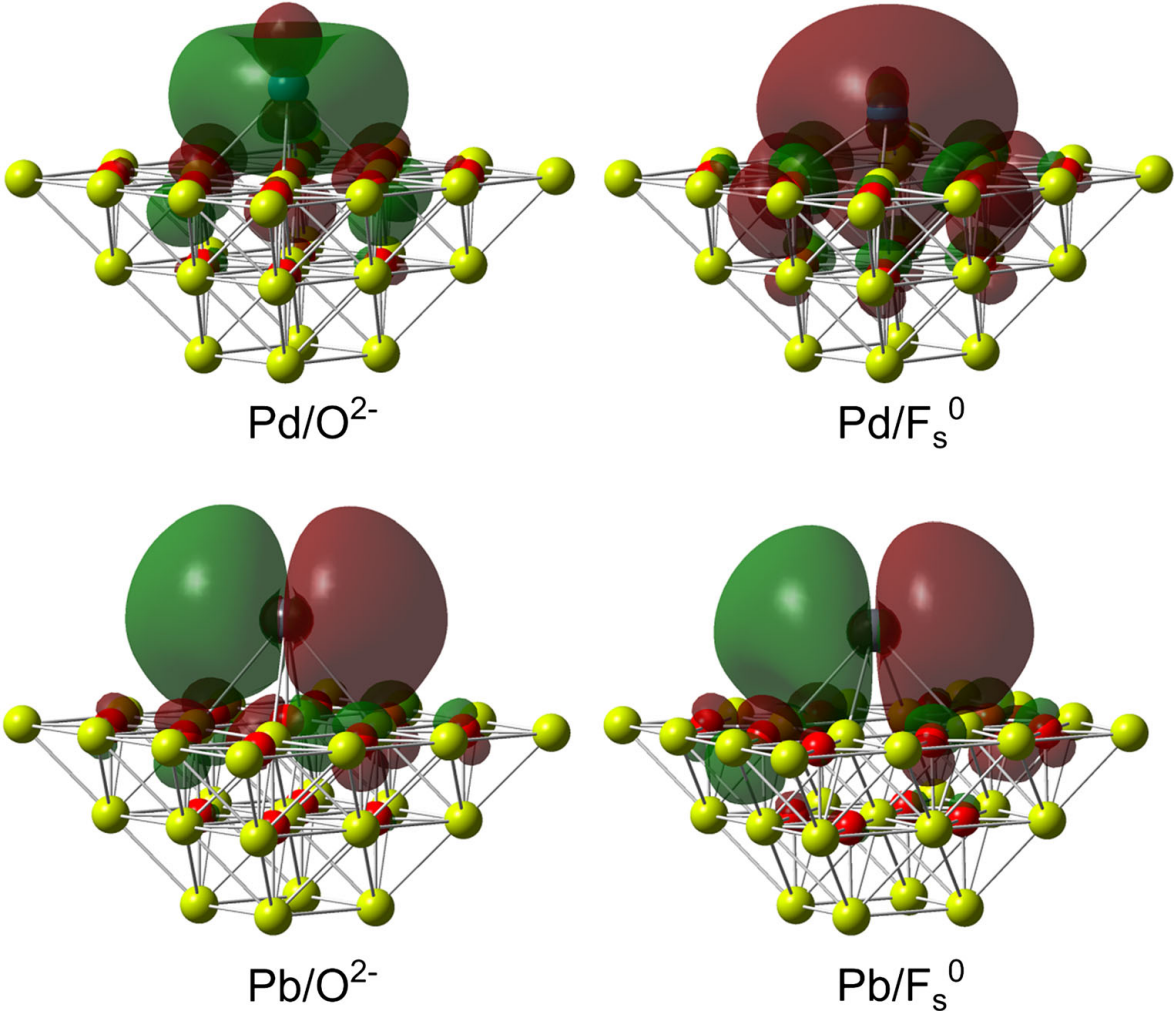
Plots of HOMO contours for Pd/O^2−^ and Pd/F_s_^0^ in their LS state and for Pb/O^2−^ and Pb/F_s_^0^ in their HS state. These contours are plotted with an isovalue of 0.01 a.u. Magnesium, oxygen, palladium and lead are colored yellow, red, blue, and gray, respectively (color figure online)

It is also interesting to examine how the surface vacancies affect the highest occupied molecular orbital (HOMO) for the Pd/F_s_^*n*+^ and Pb/F_s_^*n*+^ structures. The HOMO determines, to a certain extent, the reactivity of adsorbed metal atoms in catalytic processes. The contours of HOMO for Pd/F_s_^0^ and Pb/F_s_^0^ are plotted in Fig. 1. For comparison, the HOMO contours of Pd/O^2−^ and Pb/O^2−^ are also depicted. It can be seen that the presence of vacancy noticeably influences the shape of HOMO for Pd/F_s_^0^. The HOMO of Pd/O^2−^ consists of the dominant contribution from Pd orbitals and several minor contributions of p-type orbitals belonging to the surface O atoms of adsorption center. The part of HOMO around Pd has the characteristics of an *s*-*d*_z_^2^ hybridized orbital. For the HOMO of Pd/F_s_^0^, the share of Pd-atom *d*_z_^2^ orbital is reduced and an s-like contribution from the extra electrons of F_s_^0^ predominates. In contrast to the O^2−^ and F_s_^0^ centers occupied with the Pd atom, the Pb/O^2−^ and Pb/F_s_^0^ structures exhibit very similar shapes of their HOMOs. These HOMOs are singly occupied orbitals, because the HS state is preferred for Pb/O^2−^ and Pb/F_s_^0^. The HOMOs show the dominant contribution from the Pb-atom p orbital with its lobes parallel to the surface. The lack of any significant change in the shape of HOMO for Pb/O^2−^ and Pb/F_s_^0^ is associated with the geometries of Pb/O^2−^ and Pb/F_s_^0^. For these structures, their *h* height of Pb from the surface adopts large values that additionally are quite close to one another. The shape of HOMO for Pb/F_s_^+^ resembles that observed for the Pb/F_s_^0^ structure (see Fig. S2 in Electronic Supplementary Material).

## Conclusion

The results reported in this work point out that the presence of vacancies on the MgO(100) surface, such as F_s_^*n*+^ and V_s_^*n*−^, has an important influence on the geometric, energetic, and electronic parameters characterizing the adsorption of Pd and Pb atoms. The F_s_^0^ and F_s_^+^ vacancies, which are most likely among the F_s_^*n*+^ and V_s_^*n*−^ defects on MgO(100), constitute the centers at which the adsorption of single Pd or Pb atoms is more exothermic than at the regular O^2−^ centers. The *E*_ads_ values of Pd/F_s_^0^ and Pd/F_s_^+^ in their preferred spin states are at least 1 eV larger than the corresponding energies of Pb/F_s_^0^ and Pb/F_s_^+^. In that regard, the presence of F_s_^0^ and F_s_^+^ on MgO(100) does not change the energetic preference of Pd-atom adsorption over Pb-atom adsorption. Such preference was previously detected experimentally and is confirmed here computationally. The Pd/F_s_^0^ and Pd/F_s_^+^ structures favor the spin state with the maximum spin pairing, whereas Pb/F_s_^0^ and Pb/F_s_^+^ are most stable in their HS states. Due to its large atomic radius, the Pb atom at the F_s_^0^ and F_s_^+^ centers is adsorbed at only slightly smaller height than at the O^2−^ center. This contrasts with the large reduction of *h* in the Pd/F_s_^0^ and Pd/F_s_^+^ structures, if compared to the *h* value of Pd/O^2−^. This reduction leads to larger increases in *E*_ads_ and in the amount of electron charge transferred to the metal atom, as well as to a change in the shape of HOMO for Pd/F_s_^0^ and Pd/F_s_^+^. The Pd- and Pb-atom adsorption at the V_s_^*n*−^ vacancies, which are less abundant on MgO(100), is highly exothermic, far exceeding the *E*_ads_ energies obtained for Pd/F_s_^*n*+^ and Pb/F_s_^*n*+^. In particular, the formation of Pb/V_s_^0^ and Pb/V_s_^−^ structures is associated with extremely high *E*_ads_ energies, which turn out to be sufficient to stabilize the LS state of these structures.

The presented quantum mechanical study of the surface vacancy effect is a tentative step in elucidating the properties of Pd–Pb/MgO catalysts. The findings made for Pb/F_s_^*n*+^ and Pb/V_s_^*n*−^ may be of particular importance, because the Pb-atom adsorption on the defective MgO(100) surface has not been investigated theoretically so far.

## Methods

The structures of F_s_^*n*+^ and V_s_^*n*−^ centers with an adsorbed Pd or Pb atom were determined using a theoretical quantum mechanical approach based on the B3LYP computational method [51–53] and the embedded cluster model of surface [54]. These structures are denoted in this work by the abbreviation ‘metal atom/adsorption center’. The aforementioned computational methodology was successfully used in many previous studies of adsorption on MgO(100), e.g., [41, 55, 56]. The F_s_^*n*+^ centers were represented by two-layer [Mg_13_O_12_]^*n*+^ clusters surrounded by total ion model potentials of the nearest Mg^2+^ cations and embedded in a large array of ± 2 point charges. The V_s_^*n*−^ centers were modeled using two-layer [Mg_12_O_13_]^*n*−^ clusters and an embedding environment comprised of total ion model potentials of Mg^2+^ and an array of ± 2 point charges. The Mg and O atoms of the [Mg_13_O_12_]^*n*+^ and [Mg_12_O_13_]^*n*−^ clusters were described by the 6-31G basis set [57, 58]. Additional polarization and diffuse basis functions [58, 59] were ascribed to several Mg and O atoms directly involved in the interaction with a Pd or Pb atom. Further details of the aforementioned cluster models are given in Section S1 in Electronic Supplementary Material.

The adsorption of a single Pd or Pb atom at each investigated center was simulated by optimizing the height (*h*) of the metal atom from the surface layer of the adsorption center. The effect of surface relaxation induced by metal adsorption was also included in these calculations. Two sets of calculations differing in the kind of basis set ascribed to the metal atoms were performed to estimate basis set effects in the results of calculations. The first kind was the LANL08 basis set [60] in its LANL08(f) version for Pd and LANL08d for Pb. The def2-TZVP basis set [61] was the second kind of basis set assigned to the metal atoms. Two low-lying electronic states with different spin multiplicities were studied for the Pd- and Pb-atom adsorption. The low-spin state (LS) was characterized by the singlet multiplicity of the center with a Pd or Pb atom adsorbed, whereas the high-spin state (HS) assumed a triplet for each adsorbed metal atom.

To calculate the adsorption energy (*E*_ads_), the total energy of an adsorption center occupied with a metal atom was subtracted from a sum of the total energies of free metal atom in its ground state and the isolated surface center in its relaxed geometry. According to this definition, adsorption with a positive *E*_ads_ value is an energetically favorable (exothermic) process. The electron charge (*q*) acquired by an adsorbed Pd or Pb atom was estimated by the partial charge of the atom. This partial charge was determined according to the Bader charge analysis [62].

All calculations except the Bader charge analysis were carried out using the GAUSSIAN 09 D.01 program [63]. The Bader charge analysis was done with the Multiwfn 3.4 program [64].

## Electronic supplementary material

Below is the link to the electronic supplementary material.

Supplementary material 1 (DOC 3089 kb)
