# Supplementary material for: Effect of surface vacancies on the adsorption of Pd and Pb on MgO(100)
Source: Monatsh Chem. 2018 Feb 13;149(6):1009–15. doi: 10.1007/s00706-018-2159-1 (PMC5972179; doi:10.1007/s00706-018-2159-1)
Supplement: Supplementary file 1 — Supplementary material 1 (DOC 3089 kb) [file 706_2018_2159_MOESM1_ESM.doc]

**Electronic Supplementary Material**

for

Effect of surface vacancies on the adsorption of Pd and Pb on MgO(100)

by

Piotr Matczak

Department of Physical Chemistry, Faculty of Chemistry, University of Łódź, Pomorska 163/165, 90-236 Lodz, Poland

**S1. Further details of computational methodology**

In this work, the F_s_*^n^*^+^ and V_s_*^n^*^-^ centers (*n* = 0, 1, 2) of defective MgO(100) surface were represented using embedded cluster models. These models were based on the respective models of regular adsorption sites on the non-defective MgO(100) surface. To be precise, the models of F_s_*^n^*^+^ centers were built using the model of regular five-coordinated anionic adsorption site on the MgO(100) surface, that is, the O^2-^ center. The models of V_s_*^n^*^-^ centers were in turn constructed from the model of Mg^2+^ center corresponding to a five-coordinated Mg^2+^ cation in the surface layer of MgO(100). Below are described in detail the basic models of O^2-^ and Mg^2+^ centers first, and then the characterization of models for oxygen and magnesium vacancies is presented.

The O^2-^ center was simulated by a two-layer Mg_13_O_13_ cluster with a finite point charges (PCs) embedding. The cluster was electrically neutral and it exhibited the structure of ideal MgO crystal lattice (with its lattice constant of 4.212 Å). Its surface layer Mg_4_O_9_ had an O atom in the center, while an Mg atom occupied the center of subsurface layer Mg_9_O_4_. The surface layer was surrounded by 12 total ion model potentials (TIMPs) that were placed at the positions of Mg^2+^ cations of ideal MgO lattice. Similarly, four TIMPs were inserted at the Mg^2+^ positions beneath the subsurface layer. The Mg_13_O_13_ cluster, together with 16 TIMPs, was embedded in an array of ±2 PCs. The array consisted of 634 PCs in total and they were located at the ideal MgO lattice positions. The array was used to approximate the Madelung potential of extended surface. A it was mentioned above, 16 positive PCs near the borders of the Mg_13_O_13_ cluster were replaced by the TIMPs of Mg^2+^ cations in order to account for the finite size of these cations and to avoid spurious charge polarization. The Mg_13_O_13_ cluster plus 16 TIMPs plus the array of PCs added up to a 13 × 13 × 4 fragment of ideal MgO crystal lattice. Different basis sets were employed for the Mg and O atoms, depending on their positions in the cluster. Five O atoms in the center of the surface layer were described by the 6-31+G(d) basis set [1-3] and four nearby surface Mg atoms were treated with 6-31G(d) [1-3]. The 6-31G basis set [1,2] was ascribed to the rest of the Mg_13_O_13_ cluster. The TIMPs utilized the LANL2 effective core potential (ECP) of Mg^2+^ [4].

The geometry of the embedded Mg_13_O_13_ cluster was partially optimized to take the effect of surface relaxation into account. To be precise, the positions of five central atoms belonging to the surface layer (one O atom and four Mg atoms) were optimized. It turned out that the optimized geometry of the embedded cluster was only slightly affected by the relaxation of surface atoms. The ground state of this cluster was a singlet state.

The adsorption of a single Pd or Pb atom at the O^2-^ center was rendered as an optimization of the distance between the metal atom and the central surface O atom of the embedded Mg_13_O_13_ cluster. The metal atom was initially placed at a distance of 2.5 Å at the top of the central surface O atom. The coordinates of five central surface atoms of the embedded Mg_13_O_13_ cluster and the distance between the metal atom and the surface of the cluster were optimized. At the same time, more distant Mg and O atoms in the cluster were kept fixed.

Two kinds of ECP basis sets were used to describe the atoms of Pd and Pb. The first kind utilized the LANL08 basis set [5] in its LANL08(f) version for Pd and LANL08d for Pb. The LANL08(f) basis set employed the relativistic LANL2 pseudopotential to replace 28 inner electrons of Pd. The remaining 4*s*^2^4*p*^6^4*d*^10^ electrons were treated with a (5*s*5*p*4*d*1*f*)/[5*s*5*p*4*d*1*f*] valence basis set. The LANL08d basis set employed the relativistic LANL2 pseudopotential to replace 78 inner electrons of Pb. The remaining 6*s*^2^6*p*^2^ electrons were represented explicitly by a (3*s*4*p*1*d*)/[3*s*4*p*1*d*] valence basis set. def2-TZVP [6] was the second kind of ECP basis sets used in this work. This basis set employed the relativistic energy-consistent Stuttgart/Cologne pseudopotentials to describe 28 and 60 inner electrons of Pd and Pb atoms, respectively. The 4*s*^2^4*p*^6^4*d*^10^ electrons of Pd were treated with a (7*s*7*p*6*d*1*f*)/[6*s*4*p*3*d*1*f*] valence basis set. The 5*s*^2^5*p*^6^5*d*^10^6*s*^2^6*p*^2^ electrons of Pb were described using a (10*s*9*p*8*d*2*f*)/[6*s*5*p*3*d*1*f*] valence basis set.

The Mg^2+^ center was represented by a two-layer Mg_13_O_13_ cluster with a finite PCs embedding. Its surface layer Mg_9_O_4_ had an Mg atom in the center, while an O atom occupied the center of the subsurface layer Mg_4_O_9_. The subsurface layer was surrounded by 12 TIMPs that replaced the corresponding +2 PCs of embedding array. Beneath the subsurface layer, subsequent nine positive PCs were replaced by TIMPs. The embedding array consisted of 629 PCs. Five atoms (one Mg atom and four O atoms) in the center of surface layer were described by the 6-31+G(d) basis set and four neighboring Mg atoms were treated with 6-31G(d). The 6-31G basis set was ascribed to the rest of the Mg_13_O_13_ cluster. The geometry of the embedded cluster was partially optimized to take the effect of surface relaxation into account. The ground state of the embedded cluster was a singlet state. The adsorption of Pd or Pb at the Mg^2+^ center was simulated through optimizing the distance between a given metal atom and the central surface Mg atom of the embedded Mg_13_O_13_ cluster. The coordinates of five central surface atoms of the embedded Mg_13_O_13_ cluster were also allowed to relax in the course of the optimizations simulating the Pd- or Pb-atom adsorption. Two low-lying electronic states with different spin multiplicities were examined for the O^2-^ and Mg^2+^ centers with a single Pd or Pb atom adsorbed. The low-spin state corresponded to a singlet multiplicity, whereas the high-spin state assumed a triplet multiplicity.

The embedded cluster models representing the F_s_*^n^*^+^ centers were based on the model of O^2-^ center. The F_s_*^n^*^+^ centers were obtained by removing the central surface O atom from the embedded Mg_13_O_13_ cluster of O^2-^ center and then the appropriate charge state was imposed on the resulting embedded Mg_13_O_12_ cluster (see Figure S1a). The F_s_^0^, F_s_^+^ and F_s_^2+^ centers were formed by eliminating the central surface O, O^-^ and O^2-^ atoms or ions, respectively, from the embedded Mg_13_O_13_ cluster of O^2-^ center. Thus, the F_s_*^n^*^+^ centers were represented by the embedded [Mg_13_O_12_]*^n^*^+^ clusters. In order to provide an accurate description of these centers, the central Mg atom of the subsurface layer was described by the 6-31G basis set augmented with polarization and diffuse functions (as it is for 6-31+G(d)). The geometries of the embedded [Mg_13_O_12_]*^n^*^+^ clusters were partially optimized to take the effect of surface relaxation into account. The ground states of the embedded clusters representing the F_s_^0^, F_s_^+^ and F_s_^2+^ centers corresponded to singlet, doublet and singlet multiplicities, respectively. The coordinates of four Mg surface atoms surrounding each O vacancy and the perpendicular distance of metal atom from the vacancy were optimized during the process of metal atom adsorption. Two electronic states were considered for the F_s_*^n^*^+^ centers with the Pd or Pb atom adsorbed. The low-spin state corresponded to either a singlet (for Pd/F_s_^0^, Pd/F_s_^2+^, Pb/F_s_^0^ and Pb/F_s_^2+^) or doublet configuration (for Pd/F_s_^+^ and Pb/F_s_^+^). The high-spin state exhibited either a triplet (for Pd/F_s_^0^, Pd/F_s_^2+^, Pb/F_s_^0^ and Pb/F_s_^2+^) or quartet multiplicity (for Pd/F_s_^+^ and Pb/F_s_^+^).

The embedded cluster models representing the V_s_*^n^*^-^ centers were based on the model of Mg^2+^ center. Below only changes relative to the model of Mg^2+^ center are indicated. The V_s_^0^, V_s_^-^ and V_s_^2-^ centers corresponded to the removal of the central surface Mg, Mg^+^ and Mg^2+^ atom or ion, respectively, from the embedded Mg_13_O_13_ cluster of Mg^2+^ center. In other words, the neutral V_s_^0^ center was represented by the embedded [Mg_12_O_13_]^0^ cluster (see Figure S1b), while the singly and doubly charged magnesium vacancy centers (V_s_^-^ and V_s_^2-^) were modeled as the embedded [Mg_12_O_13_]^-^ and [Mg_12_O_13_]^2-^ clusters, respectively. The 6-31+G(d) basis set was assigned to the central O atom of the subsurface layer. The ground states of the embedded clusters representing the V_s_^0^, V_s_^-^ and V_s_^2-^ centers showed triplet, doublet and singlet multiplicities, respectively. The coordinates of four O surface atoms surrounding each Mg vacancy and the distance between the metal atom and the vacancy were optimized when the metal atom was placed at the top of vacancy. The low-spin state of Pd/V_s_*^n^*^-^ and Pb/V_s_*^n^*^-^ corresponded to either a singlet (for Pd/V_s_^0^, Pd/V_s_^2-^, Pb/V_s_^0^ and Pb/V_s_^2-^) or doublet configuration (for Pd/V_s_^-^ and Pb/V_s_^-^). Their high-spin state exhibited either a triplet (for Pd/V_s_^0^, Pd/V_s_^2-^, Pb/V_s_^0^ and Pb/V_s_^2-^) or quartet multiplicity (for Pd/V_s_^-^ and Pb/V_s_^-^).


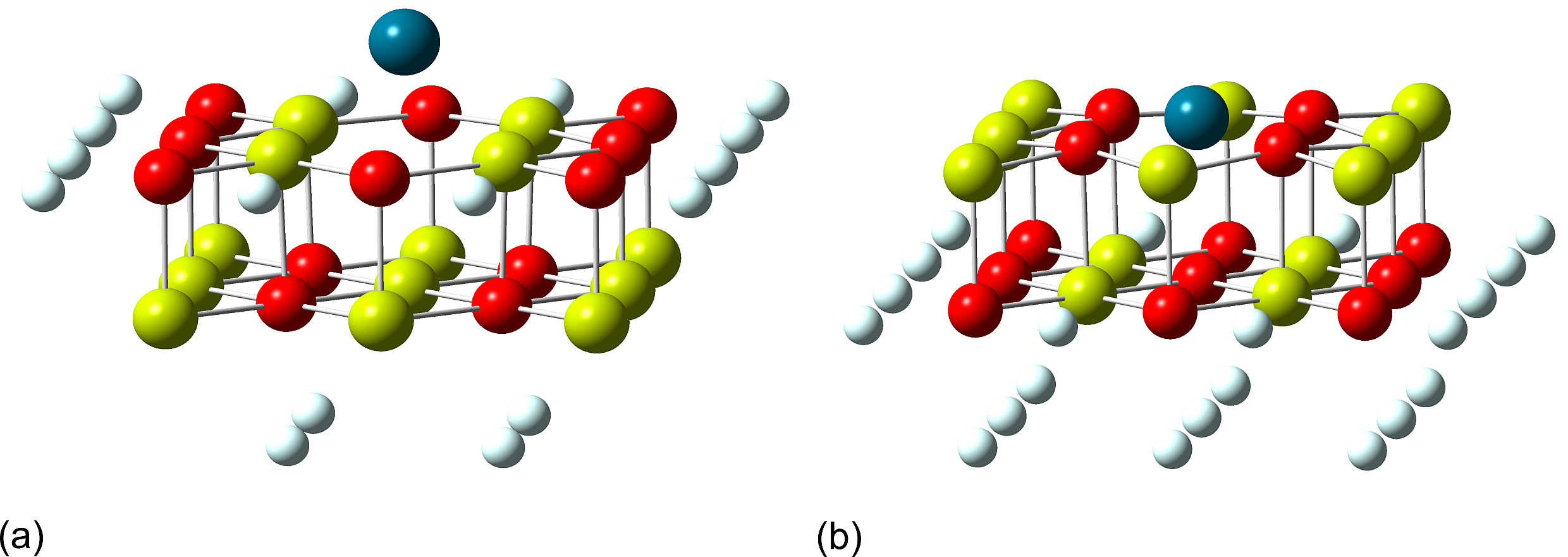


Figure S1. (a) The Mg_13_O_12_ cluster representing the F_s_*^n^*^+^ centers and (b) the Mg_12_O_13_ cluster used to model the V_s_*^n^*^-^ centers. A Pd atom adsorbed at these centers is presented as an example. TIMPs surrounding the clusters are shown but the array of PCs is hidden. Magnesium, oxygen, palladium, and outer Mg^2+^ described by TIMPs are colored yellow, red, dark blue, and light blue, respectively.

**S2. Additional figures**

**
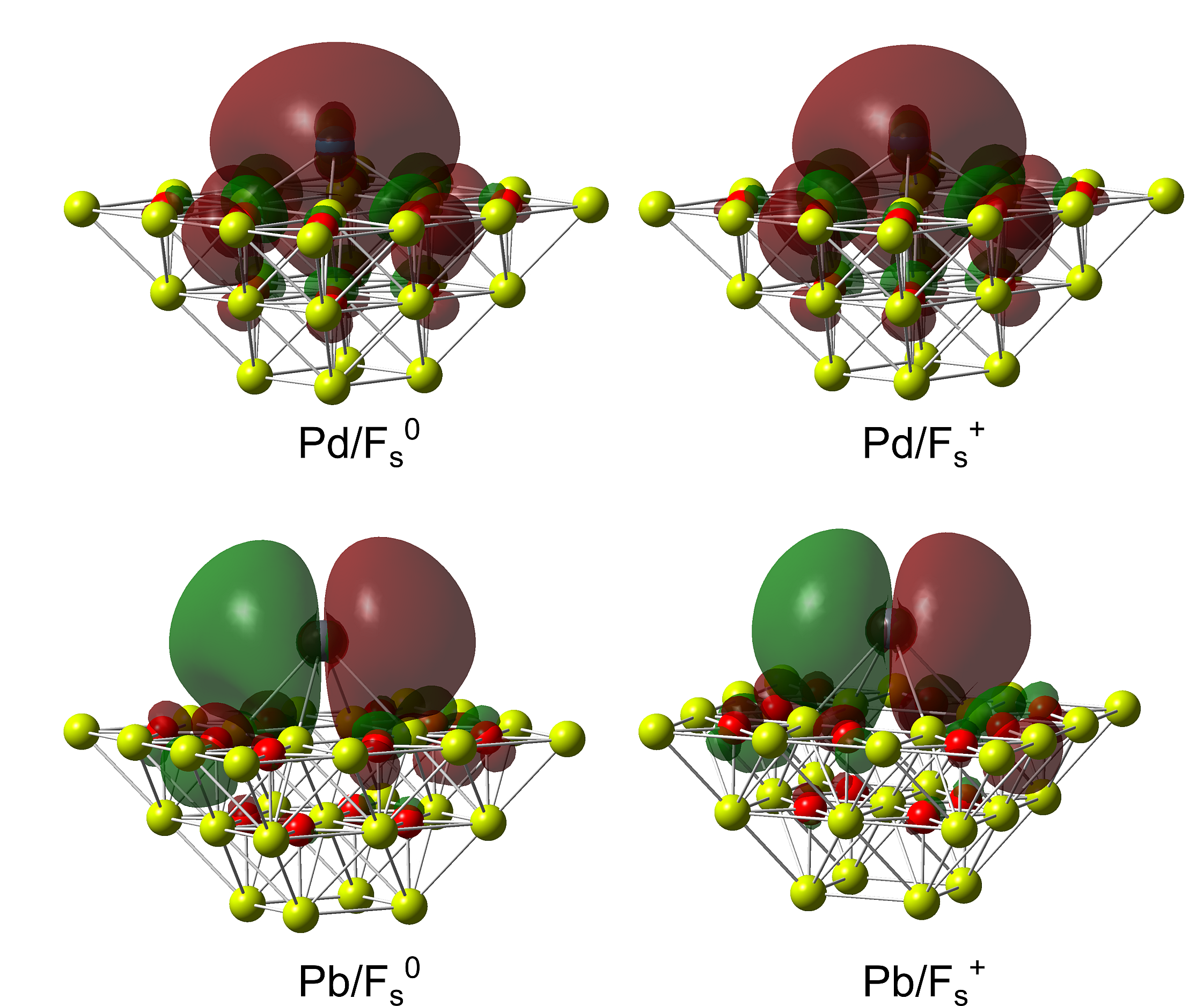
**

Figure S2. Plots of HOMO contours for Pd/F_s_^0^ and Pd/F_s_^+^ in their LS state and for Pb/F_s_^0^ and Pb/F_s_^+^ in their HS state. These contours are plotted with an isovalue of 0.01 a.u. Magnesium, oxygen, palladium and lead are colored yellow, red, blue, and gray, respectively.

**References**

1. Hehre WJ, Ditchfield R, Pople JA (1972) Self-consistent molecular orbital methods. XII. Further extensions of Gaussian-type basis sets for use in molecular orbital studies of organic molecules. J Chem Phys 56:2257–2261

2. Francl MM, Pietro WJ, Hehre WJ, Binkley JS, Gordon MS, DeFrees DJ, Pople JA (1982) Self‐consistent molecular orbital methods. XXIII. A polarization‐type basis set for second‐row elements. J Chem Phys 77:3654–3665

3. Clark T, Chandrasekhar J, Spitznagel GW, Schleyer PvR (1983) Efficient diffuse function-augmented basis sets for anion calculations. III. The 3-21+G basis set for first-row elements, Li–F. J Comput Chem 4:294–301

4. Wadt WR, Hay PJ (1985) Ab initio effective core potentials for molecular calculations. Potentials for main group elements Na to Bi. J Chem Phys 82:284–298

5. Roy LE, Hay PJ, Martin RL (2008) Revised basis sets for the LANL effective core potentials. J Chem Theory Comput 4:1029–1031

6. Weigend F, Ahlrichs R (2005) Balanced basis sets of split valence, triple zeta valence and quadruple zeta valence quality for H to Rn: design and assessment of accuracy. Phys Chem Chem Phys 7:3297–3305
